# Supplementary material for: New Benzo[h]quinolin-10-ol Derivatives as Co-sensitizers for DSSCs
Source: Materials (Basel). 2021 Jun 18;14(12):3386. doi: 10.3390/ma14123386 (PMC8234456; doi:10.3390/ma14123386)
Supplement: Supplementary file 1 [file materials-14-03386-s001.zip › materials-1238556-supplementary.pdf]

Supplementary

New benzo[h]quinolin-10-ol Derivatives as Co-sensitizers for DSSCs

Aneta Slodek [1], Paweł Gnida [2], Jan Grzegorz Małecki [1], Grażyna Szafraniec-Gorol [1], Pavel Chulkin [3], Marharyta Vasylieva [2], Jacek Nycz [1], Marcin Libera [1] and Ewa Schab-Balcerzak [1,2,*]

[1] Institute of Chemistry, University of Silesia, 9 Szkolna Str., 40-006 Katowice, Poland; a.slodek@wp.pl or aneta.slodek@us.edu.pl (A.S.), grazyna.szafraniec-gorol@us.edu.pl (G. S-G.), jan.malecki@us.edu.pl (J.G.M.), jacek.nycz@us.edu.pl (J.N.), marcin.libera@us.edu.pl (M.L.)
[2] Centre of Polymer and Carbon Materials, Polish Academy of Sciences, 34 M. Curie-Skłodowska Str., 41-819 Zabrze, Poland; pgnida@cmpw-pan.edu.pl (P.G.), mvasylieva@cmpw-pan.edu.pl (M.V.)
[3] Faculty of Chemistry, Silesian University of Technology, 9 Strzody Str., 44-100 Gliwice, Poland; pavel.chulkin@polsl.pl (P.Ch.)
* Correspondence: ewa.schab-balcerzak@us.edu.pl or eschab-balcerzak@cmpw-pan.edu.pl (E.S.-B.)

Materials

Fluorine doped tin oxide coated glass slides (FTOs, 7 Ω/sq, Sigma-Aldrich), 18NR-T Titania Paste (Greatcell Solar Materials), surfactant (Hellmanex III, Hellma Analytics), isopropanol (IPA, POCH), Ru(II)(2,2′-bipyridyl-4,4′-dicarboxylic-acid) (2,2′-bipyridyl-4,4′-ditetrabutylammonium-carboxylate) (NCS)2 (N719) and EL-HSE electrolyte were purchased from Sigma Aldrich. Ethanol (Avantor Performance Materials), N,N-dimethylformamide (Aldrich), chenodeoxycholic acid (Sigma) were used to devices preparation.

Measurements

The NMR spectra were recorded on a Bruker Avance 400 MHz instrument by using DMSO-d_6 as a solvent. The steady-state emission and excitation spectra were measured for methanol (MeOH) and dimethylformamide (DMF) solutions on the FLS-980 spectrophotometer at ambient temperature using 450 W Xe arc lamp as a light source and PMT (Hamamatsu, R928P) in cooled housing as a detector. The quantum yields of fluorescence were determined by the absolute method at room temperature, using the integrating sphere with solvent as a blank. Compounds are excited in the wavelength corresponding to the absorption wavelength of the compounds. The time-resolved measurement has been prepared at optically diluted solutions at room temperature using the time-correlated single photon counting methods on the FLS-980 spectrophotometer. Excitation wavelengths were obtained using the picosecond pulsed diode EPLED–280 and 340 nm with 100 ns pulse period as light sources. PMT (Hamamatsu, R928P) in cooled housing was used as a detector. The system was aligned at emission wavelengths.

Additionally, for the analysis of fluorescence decay, an instrument response function needs to be obtained. The IRF contains information about the time response of the overall optical and electronic system. The IRF was designed using LUDOX solution as a standard at 340 nm or 280 nm. The influences of the Raman scattering of solvent on the sample's emission were avoided utilizing a filter. The IRF contains information about the time response of the overall optical and electronic system. Cyclic voltammetry measurements were conducted in 0.1 M solutions of Bu_4PF_6, 99% in N, N-dimethylformamide (1.0 mM concentration) at room temperature at a potential rate of 50 mV/s and were calibrated against a ferrocene/ferrocenium redox couple. The electrochemical cell comprises the platinum electrode with a 1 mm diameter of Pt as a working electrode, an $Ag|Ag^+$ electrode as a pseudoreference electrode and a platinum coil as an auxiliary electrode. The morphology of the electrodes' surface in nanoscale was characterized by atomic force microscopy (AFM) using TopoMetrix Explorer device, operating in contact mode, in air, in constant force regime. The thickness and morphology in a broad range of TiO_2 layers

were determined using optical microscope OLYMPUS DSX510. The cross-sectional SEM images were taken using the SEM microscope Quanta/FEG 250/FEI Company. The photovoltaic parameters were determined by a PV Solutions Solar Simulator and a Keithley 2400 (under AM 1.5 G illumination 100 mW/cm^2) (Tektronix, Inc., Beaverton, OR, USA). Impedance spectra of the photovoltaic cells were registered using BioLogic SP-150 Potentiostat. During the measurement, the samples were exposed to the light of illuminance (1000 W/m^2) produced by PV Solutions Solar Simulator. Impedance spectra were obtained in 100 kHz – 0.1 Hz frequency range with a resolution of 10 points per decade in logarithmic scale (total number of frequencies in one spectrum was 60), AC voltage amplitude was 10 mV. The applied voltage was changed in a range from 0 V to the open-circuit voltage of the device with an increment of 0.05 V in staircase mode. Analysis of electrochemical impedance spectra and determination of equivalent circuit parameters was accomplished using EIS spectrum analyzer software (G.A. Ragoisha, A.S. Bondarenko, http://www.abc.chemistry.bsu.by/vi/analyser).

DSSC Preparation

The FTOs slides were washed in a solution of Hellamanex III and deionised water (1:9, v/v) for 5 minutes, next then rinsed twice with hot deionised water. After that, the FTOs substrates were placed in an ultrasonic bath in IPA and again rinsed twice by deionised water. Then, all of the FTOs substrates were dried in the air. The TiO_2 layers were screen-printed on previously cleaned FTOs. The next step was to heat the substrates covered by TiO_2 layers at 500 ^{0}C in the air for 30 min. The prepared TiO_2 substrates on FTOs glasses were immersed in N719, **1a**, **2a** or N719+**1a**/**2a** solutions (c= 3x10^{-4} M) of DMF, co adsorbent (CDCA) was added to selected solutions. After 24 h, excess of dyes was flush by EtOH. The fabricated photoanodes were employed to assembly sandwich-type devices with structure FTO/TiO_2+dye/EL-HSE/Pt/FTO. The counter-electrode was nanoplatin applied on FTO glass. The liquid electrolyte consists of iodide/triiodide redox couple was placed between electrodes.

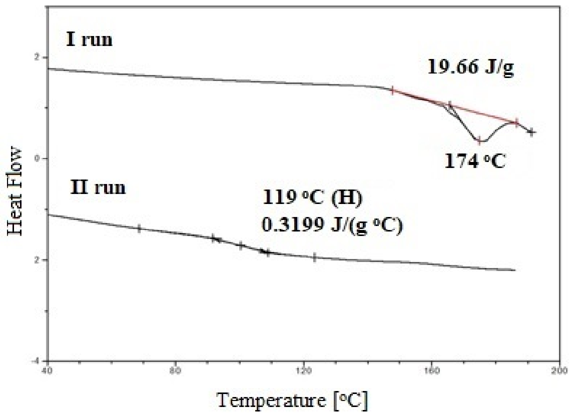

Figure S1. DSC thermograms of the synthesized cyanoacrylic acids 10-hydroxobenzo- [h]quinoline derivative with two anchoring units (**2a**).

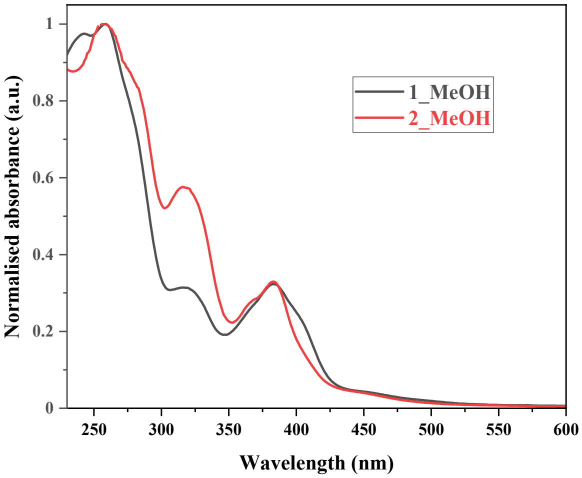

Figure S2. Absorption spectra of aldehydes **1** and **2** recorded in the MeOH solution ($c = 10^{-5}$ mol/L).

Table S1. Electronic spectral data for aldehydes **1** and **2** recorded in methanol solution.

	**λ_{max} (nm) (ε ($M^{-1}cm^{-1}$))**	**PL λ_{em} (nm)**	**E_g^{OPT} (eV)**
**1**	243(12882), 258(13500), 317(4308) 383(4505)	550	2.25
**2**	222(21500), 259(26105), 317(1108), 383(208)	550	2.25

$E_g^{OPT}=1240/\lambda$

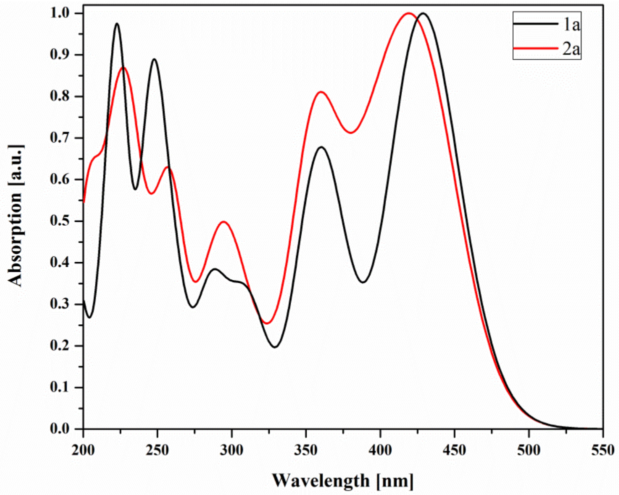

Figure S3. DFT calculated in DMF UV-vis spectra of benzo[*h*]quinolin-10-ol derivatives.

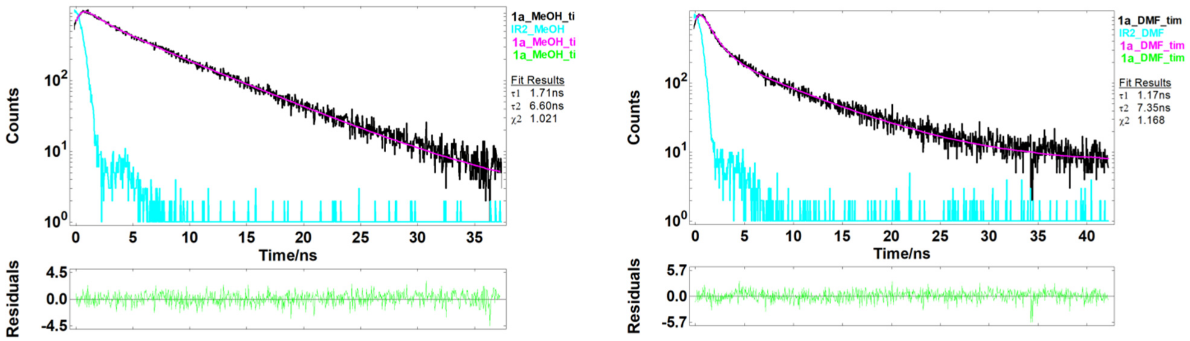

1a in MeOH **1a** in DMF

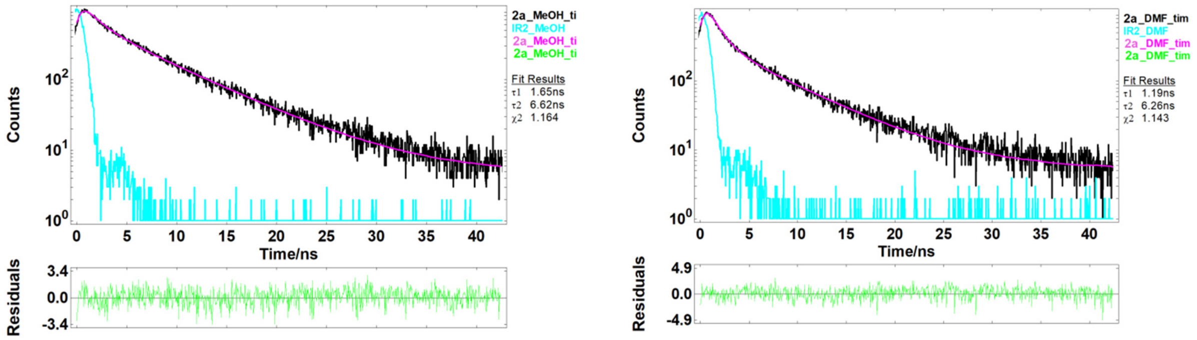

2a in MeOH **2a** in DMF

Figure S4. Fluorescence decays of **1a** and **2a** recorded in methanol (MeOH) and dimethylformamide (DMF).

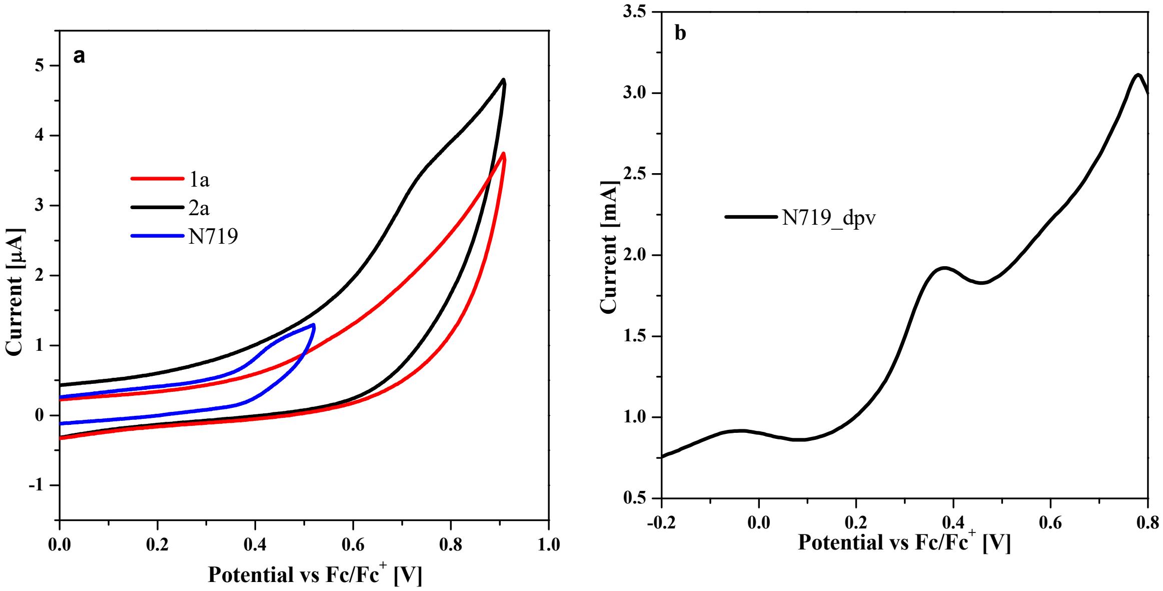

Figure S5. (**a**) CV curves of **1a**, **2a** and N719, (**b**) DPV of N719 in 0.1M Bu_4NPF_6/DMF.

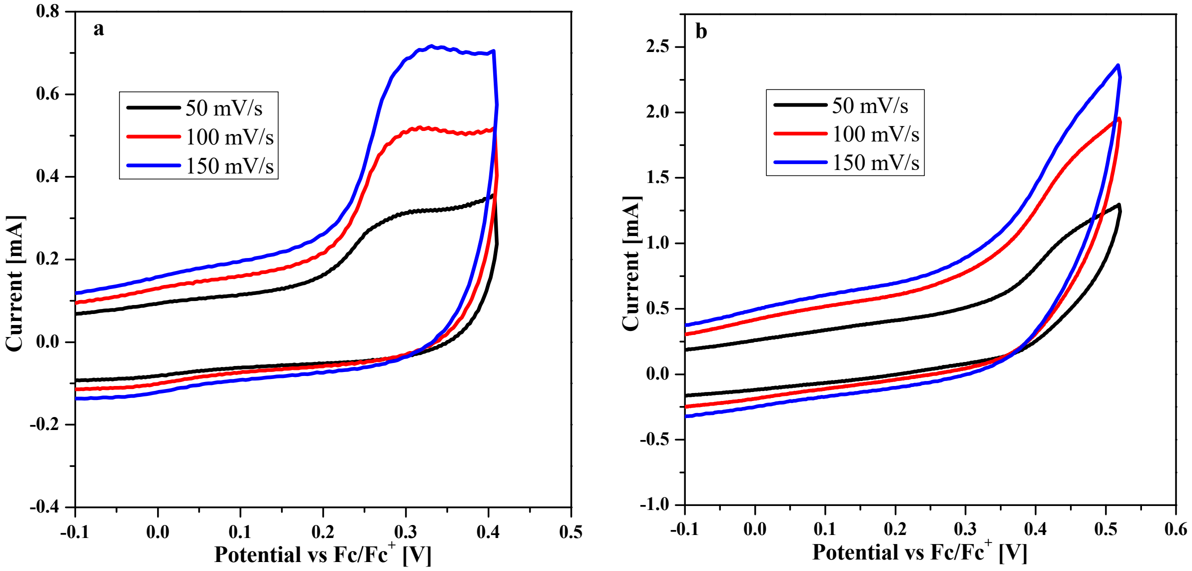

Figure S6. CV curves of N719 solution in (a) acetonitrile and (b) DMF with different scan rates.

Table S2. Electrochemical data for N719 recorded in 0.1M Bu_4NPF_6 in ACN and DMF.

	E_{ox} (V)	HOMO (eV)
ACN	0.22	−5.32
DMF	0.33	−5.43

Table S3. HOMO and LUMO contours of the $Ti_{21}O_{54}H_{24}$ cluster, CA, CDCA, DCA and adsorbed *enol*–forms of the co-adsorbents on $Ti_{21}O_{54}H_{24}$ cluster.

	HOMO	LUMO
$Ti_{21}O_{54}H_{24}$	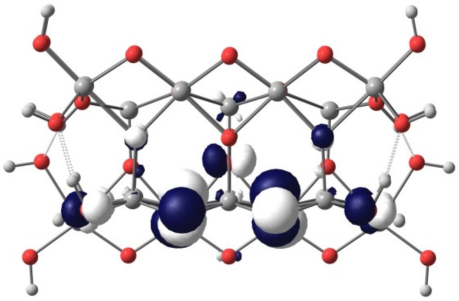	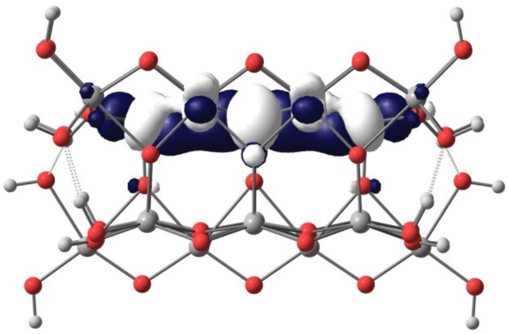

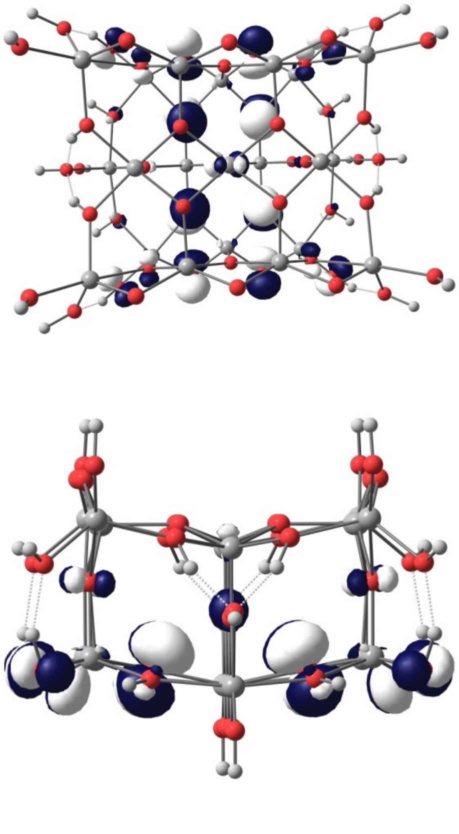

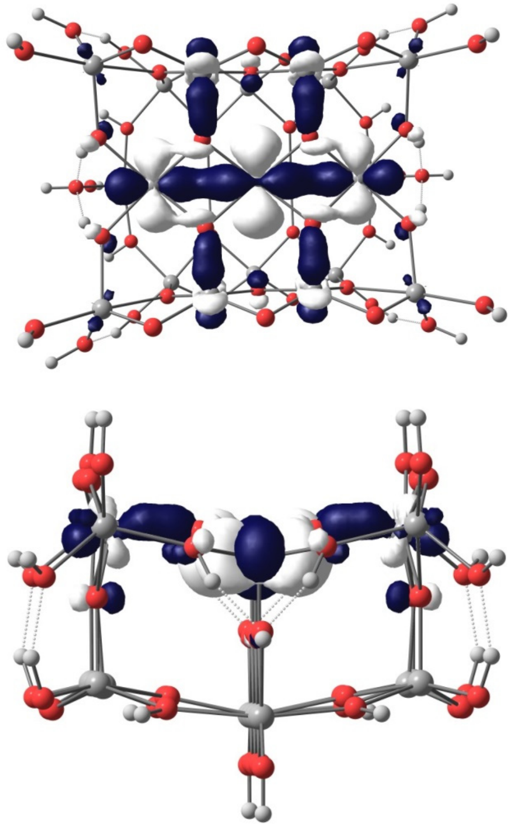

=8.27 D –6.50 eV –4.67 eV

CA

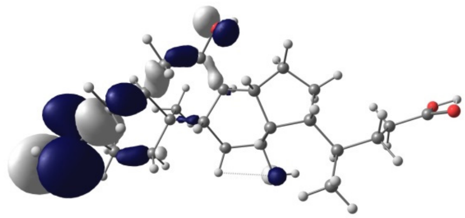

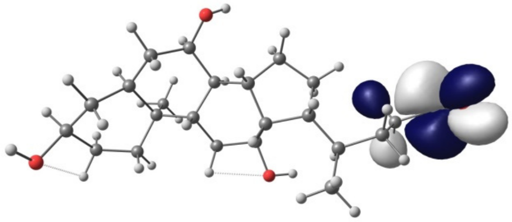

=2.78 D –5.63 eV –0.65 eV

CDCA

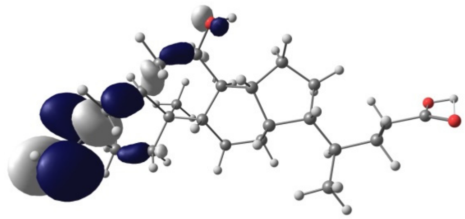

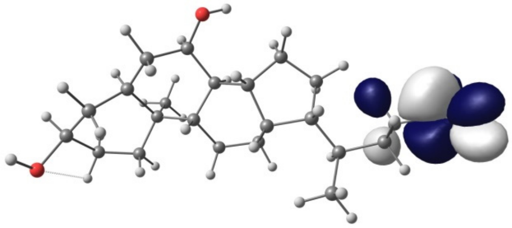

=2.07 D –5.66 eV –0.77 eV

DCA

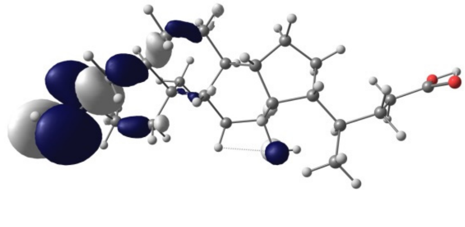

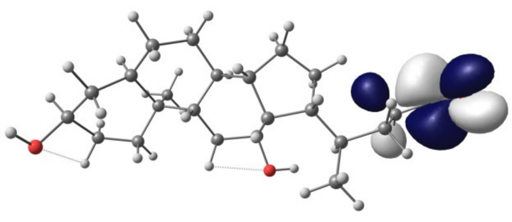

=2.68 D –5.61 eV –0.85 eV

$Ti_{21}O_{54}H_{24}$
···CA

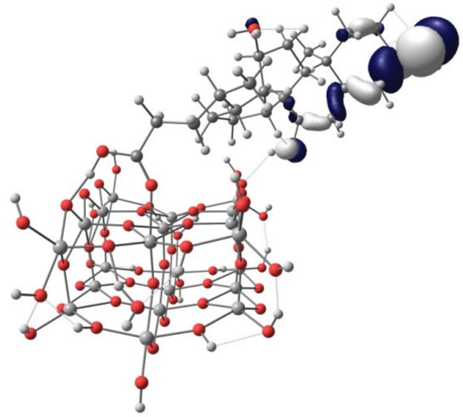

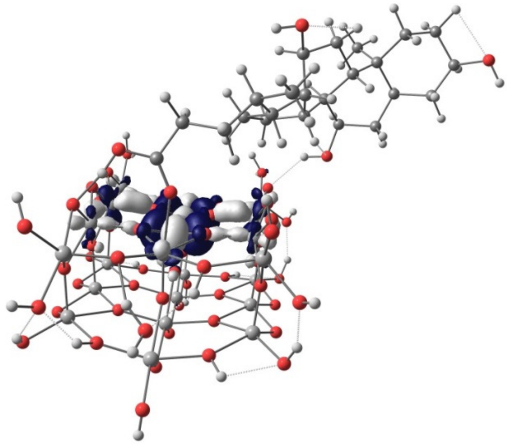

=6.88 D –5.60 eV –4.60 eV

$Ti_{21}O_{54}H_{24}$
···CDCA

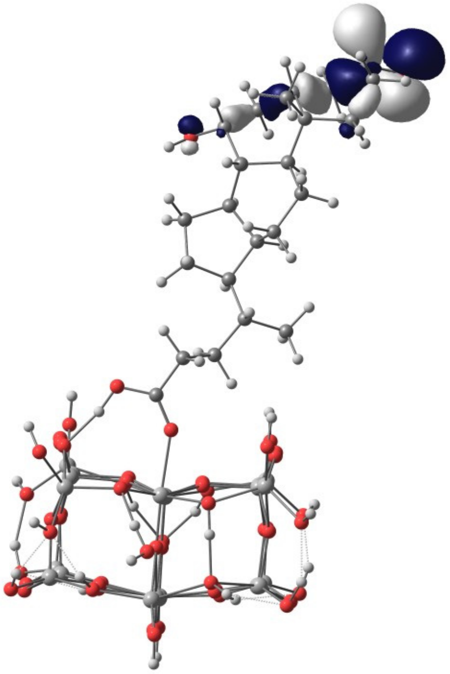

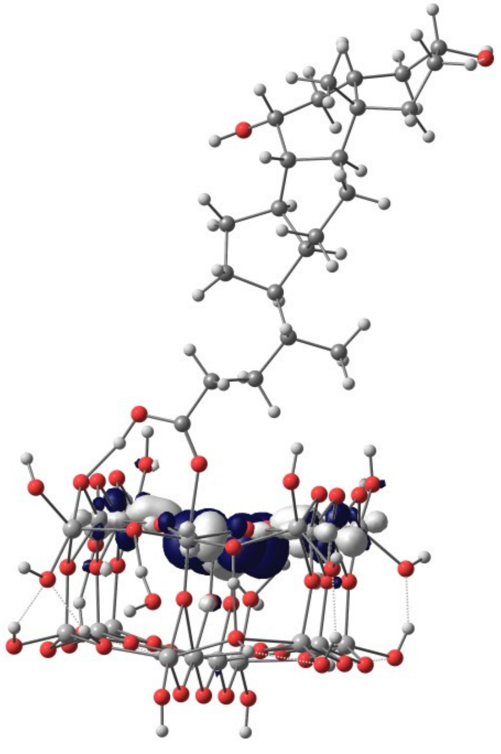

=10.44 D –5.75 eV –4.49 eV

$Ti_{21}O_{54}H_{24}$
···DCA

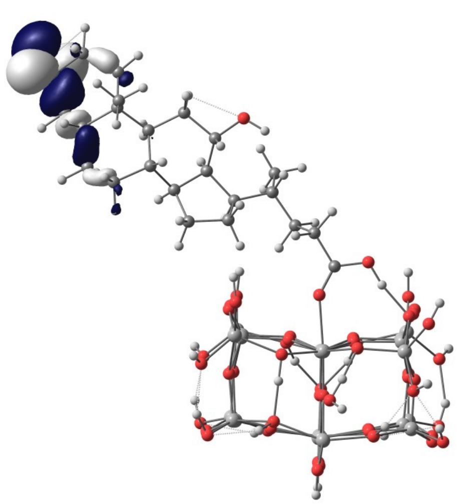

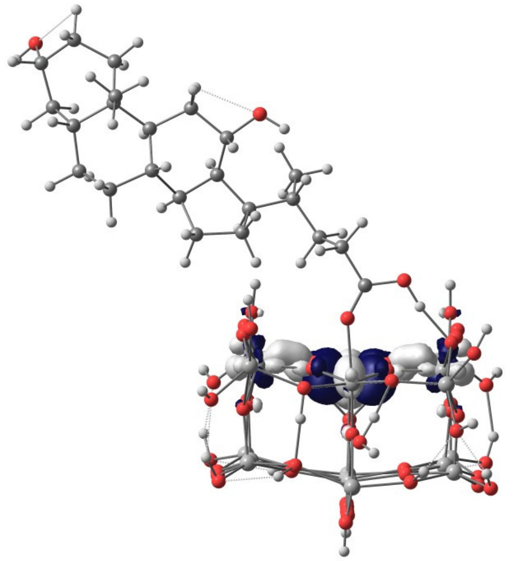

=7.84 D   –5.69 eV   –4.51 eV

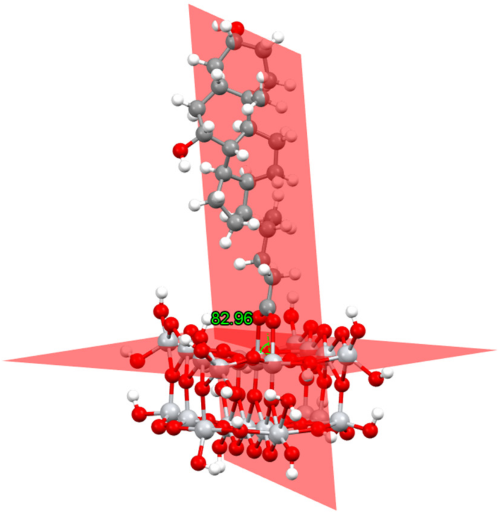

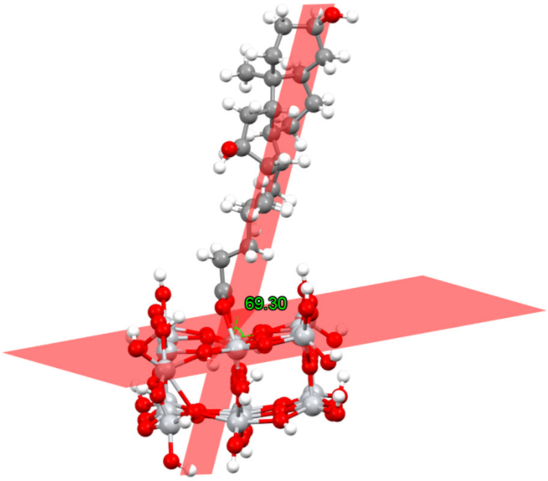

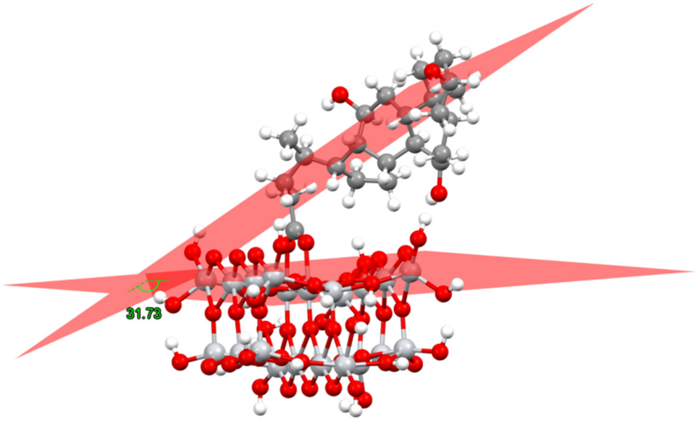

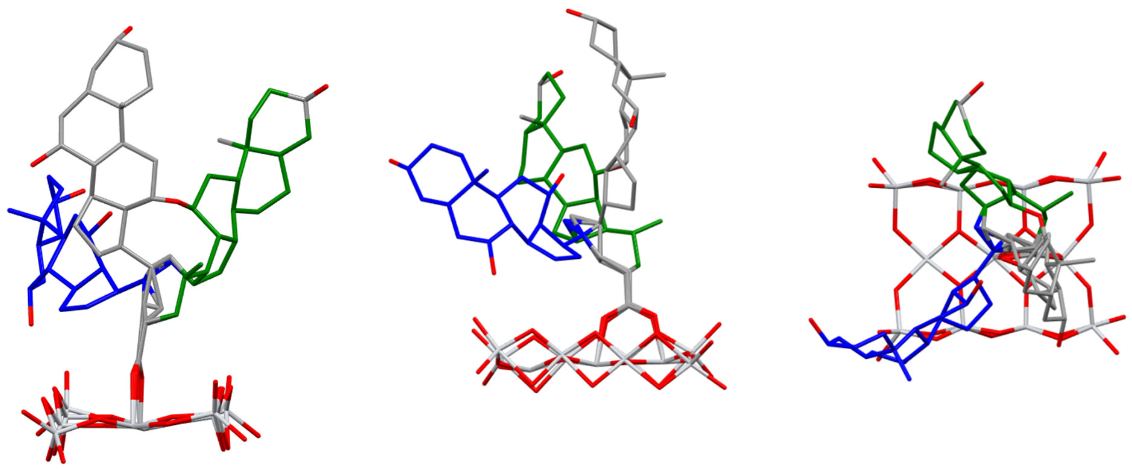

Figure S7. Angles between means planes of the CDCA, DCA and CA adsorbed on the $Ti_{21}O_{54}H_{24}$ cluster. Below front, side and top view of the surface with adsorbed molecules of all dyes (CA – blue, CDCA – grey, DCA – green).

Table S4. Adsorption of *enol*– forms of the dyes on $Ti_{21}O_{54}H_{24}$ cluster (The Gibbs free energies *G* of the species were calculated at 298.15K).

$Ti_{21}O_{54}H_{24}$···CA	$Ti_{21}O_{54}H_{24}$···CDCA	$Ti_{21}O_{54}H_{24}$···DCA
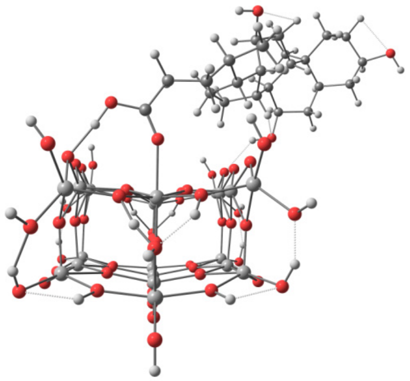	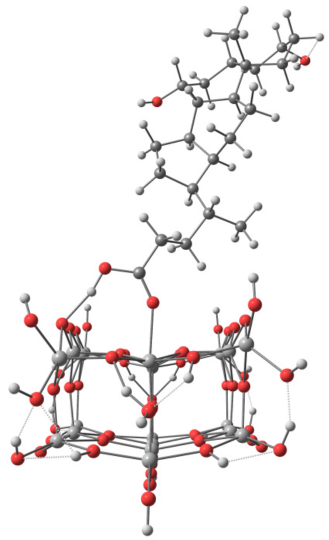	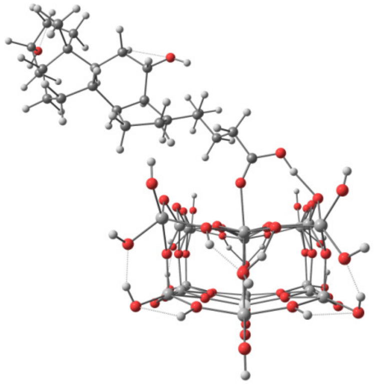
G_{ads}= –13,89 kcal/mol	G_{ads}= –17,41 kcal/mol	G_{ads}= –16.79 kcal/mol
E_{ads}= –32.82 kcal/mol	E_{ads}= –34.58 kcal/mol	E_{ads}= –35.18 kcal/mol

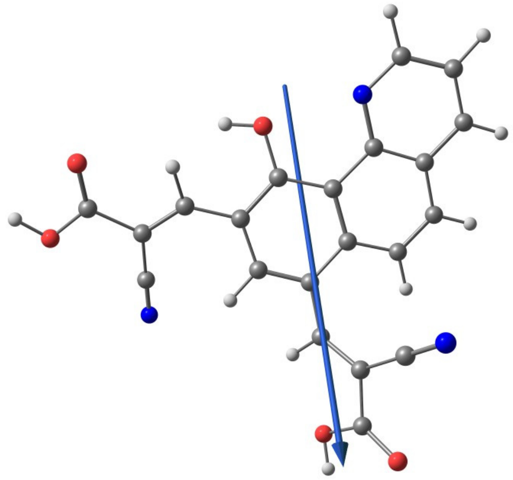

2a =4.80 D

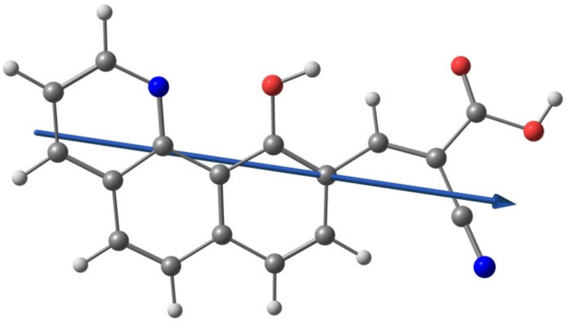

1a =4.62 D

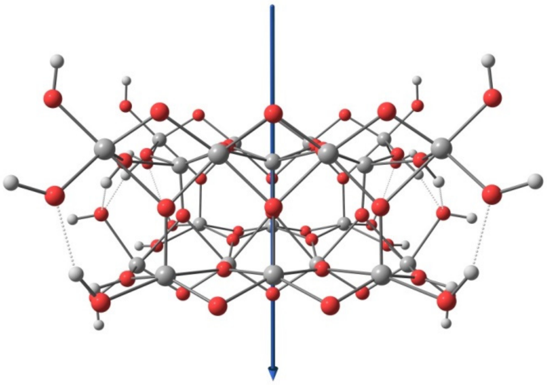

$Ti_{21}O_{54}H_{24}$ =8.52 D

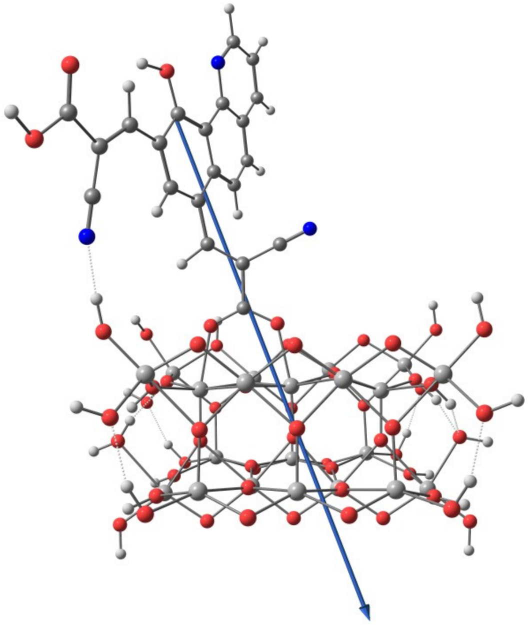

2a-a@$Ti_{21}O_{54}H_{24}$ = 11.84 D

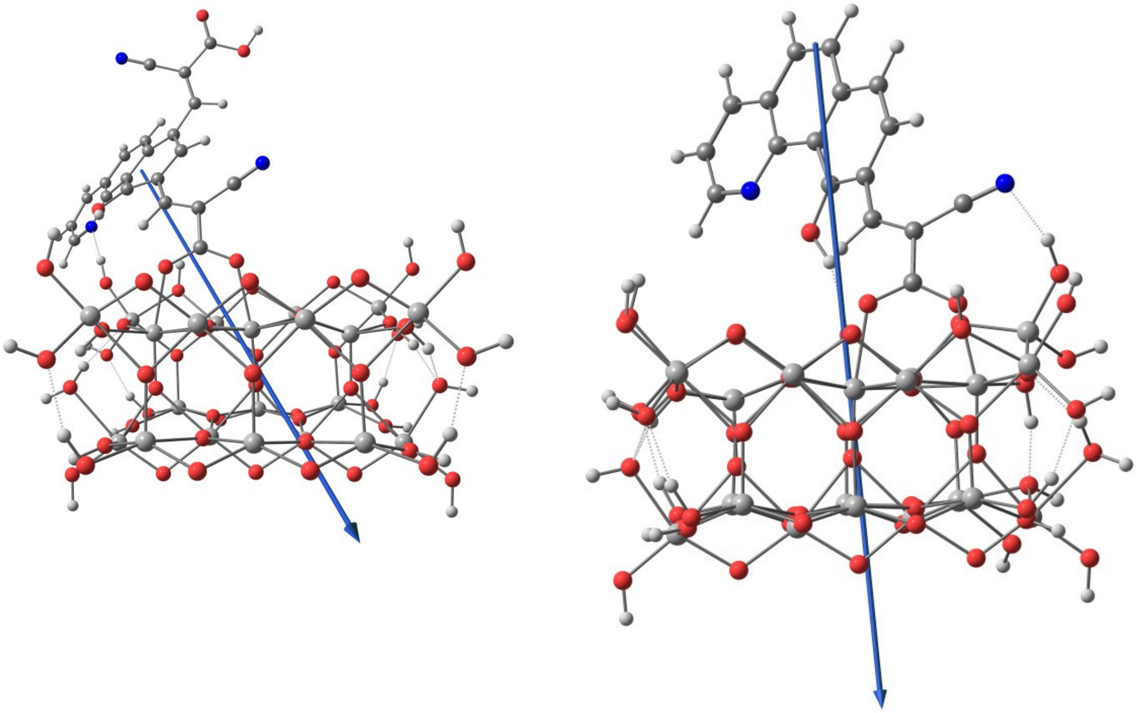

2a-b@$Ti_{21}O_{54}H_{24}$ = 11.75 D

1a@$Ti_{21}O_{54}H_{24}$ = 9.90 D

Figure S8. Calculated dipole moments of $Ti_{21}O_{54}H_{24}$ surface, **1a**, **2a** dyes and **2a-a**@$Ti_{21}O_{54}H_{24}$, **2a-b**@$Ti_{21}O_{54}H_{24}$, **1a**@$Ti_{21}O_{54}H_{24}$ systems.

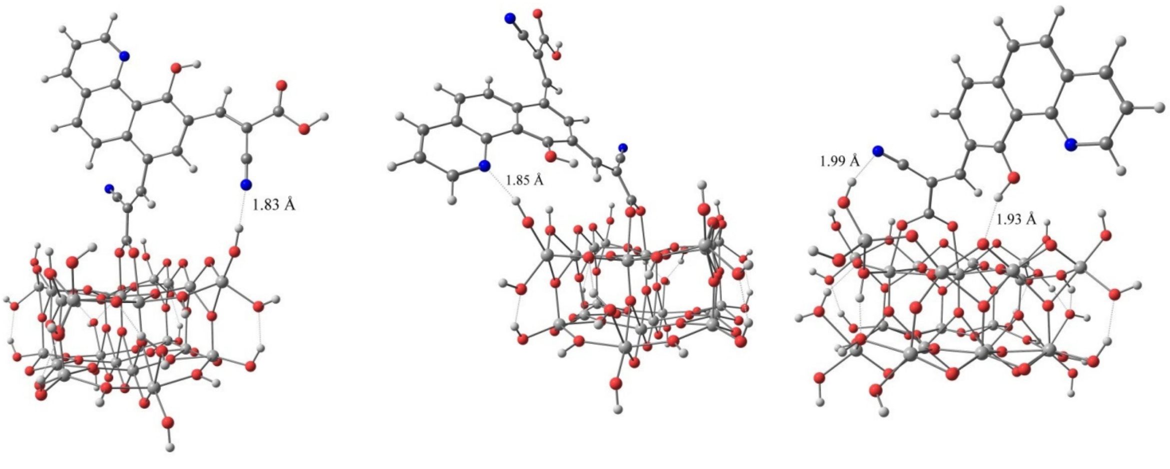

$Ti_{21}O_{54}H_{24}$···**2a-a**

$Ti_{21}O_{54}H_{24}$···**2a-b**

$Ti_{21}O_{54}H_{24}$···**1a**

Figure S9. Hydrogen bonds in the $Ti_{21}O_{54}H_{24}$···**1a/2a** systems.

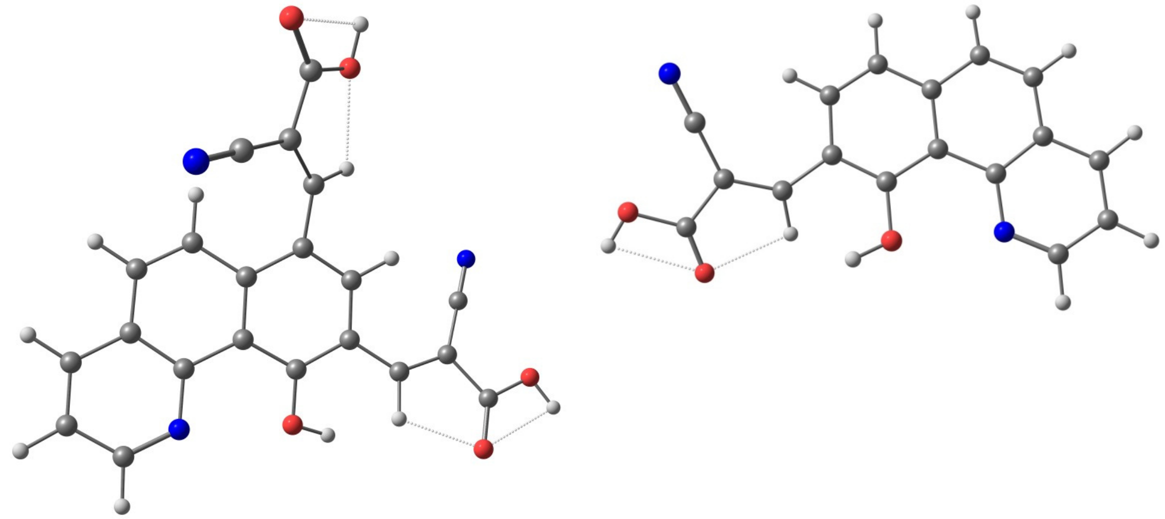

2a **1a**

Figure S10. Optimized geometries of **1a** and **2a** molecules.

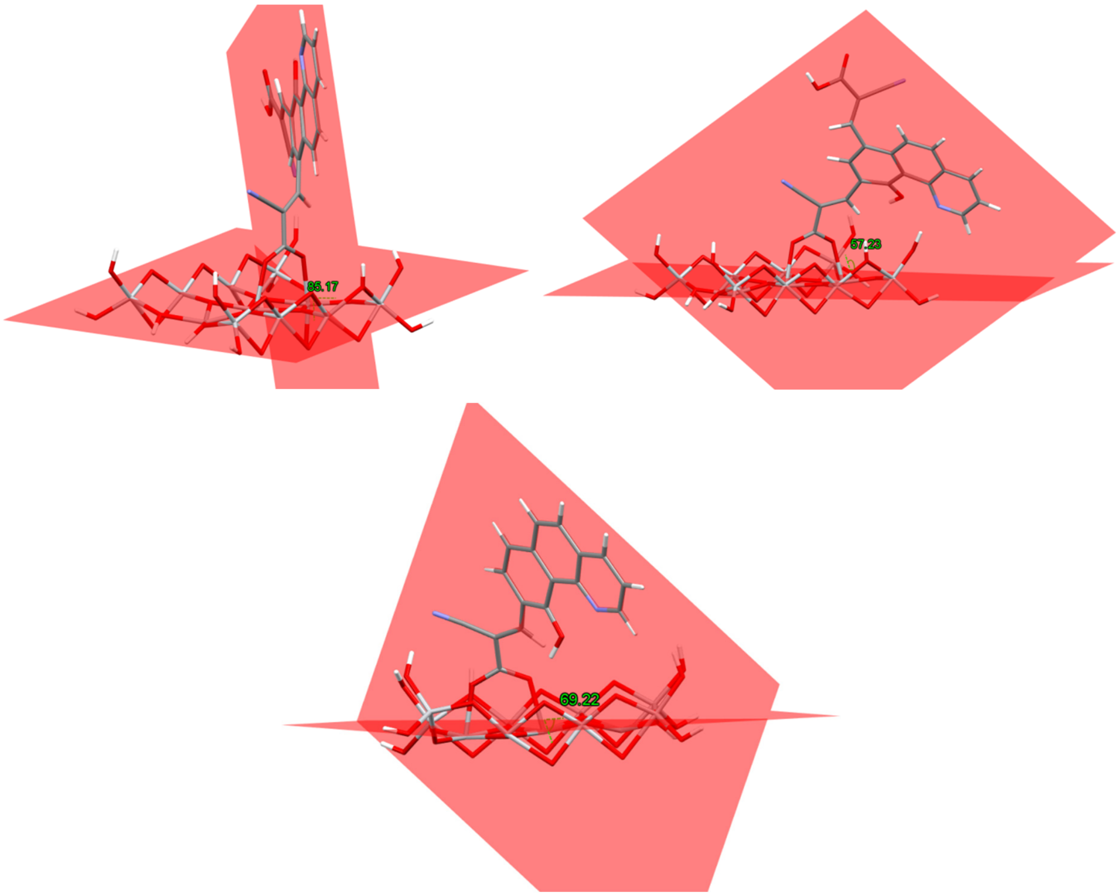

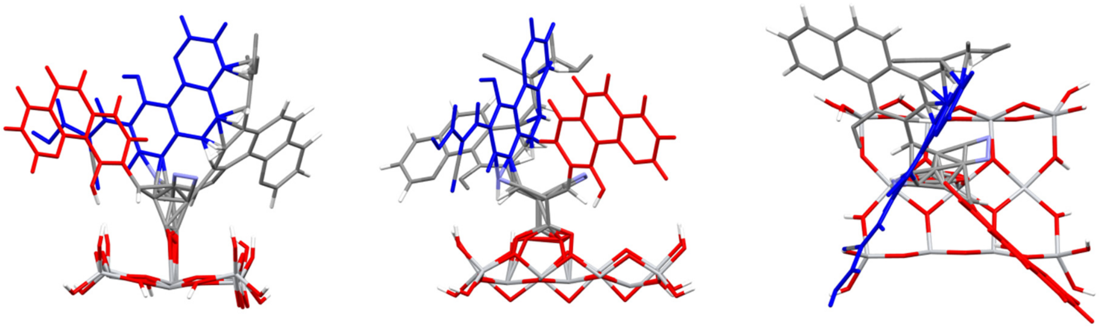

Figure S11. Angles between mean planes of the aromatic parts of **2a-a**, **2a-b** and **1a** adsorbed on the $Ti_{21}O_{54}H_{24}$ cluster. Below front, side and top view of the surface with adsorbed molecules of all dyes (**2a-a** – red, **2a-b** – grey, **1a** – blue).

Table S5. Angles between planes of benzoquinoline ring and 2-cyanoacrylic acid moiety free and adsorbed molecules of **1a**, **2a** (first column) and between planes of adsorbed dyes and surface of TiO_2 (second column).

	∢(°)	∢(°)
**2a-a***	47.99	
**2a-b***	18.56	
**1a**	17,37	
**2a-a**@$Ti_{21}O_{54}H_{24}$	53.32	85.17
**2a-b**@$Ti_{21}O_{54}H_{24}$	36.86	57.23
**1a**@$Ti_{21}O_{54}H_{24}$	33.65	69.22

* "a" refers to a cyanoacrylic substituent at position 7 and "b" to substituent in positon 9 in benzoquinoline

HOMO LUMO

$Ti_{21}O_{54}H_{24}$

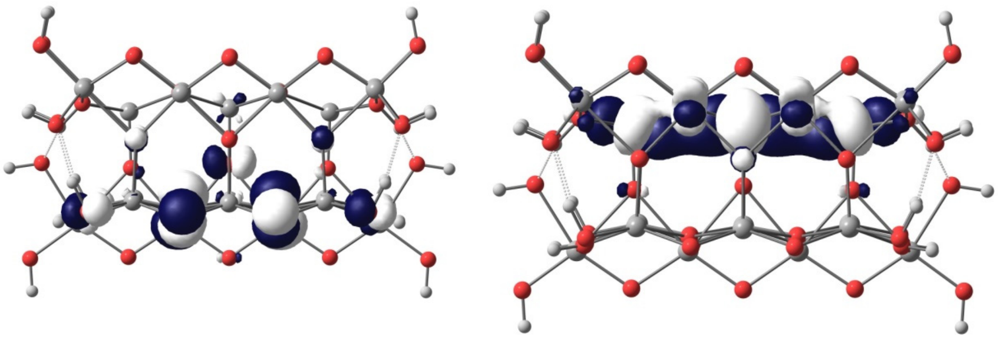

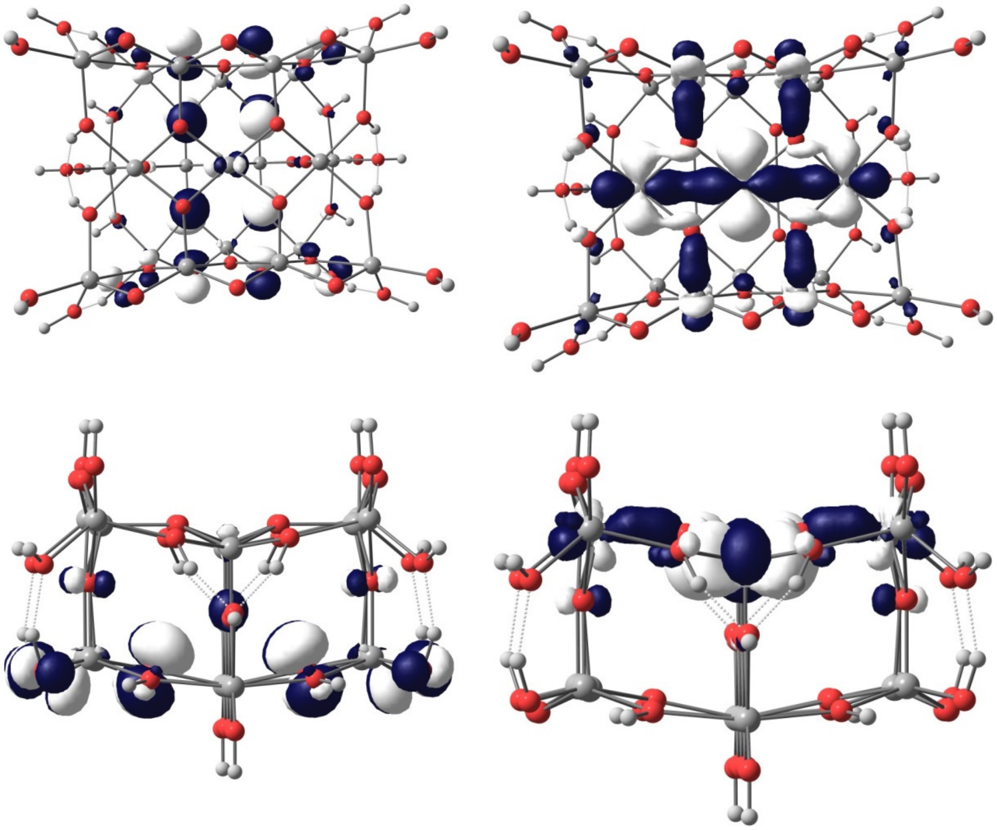

–7.61 eV –3.90 eV

2a

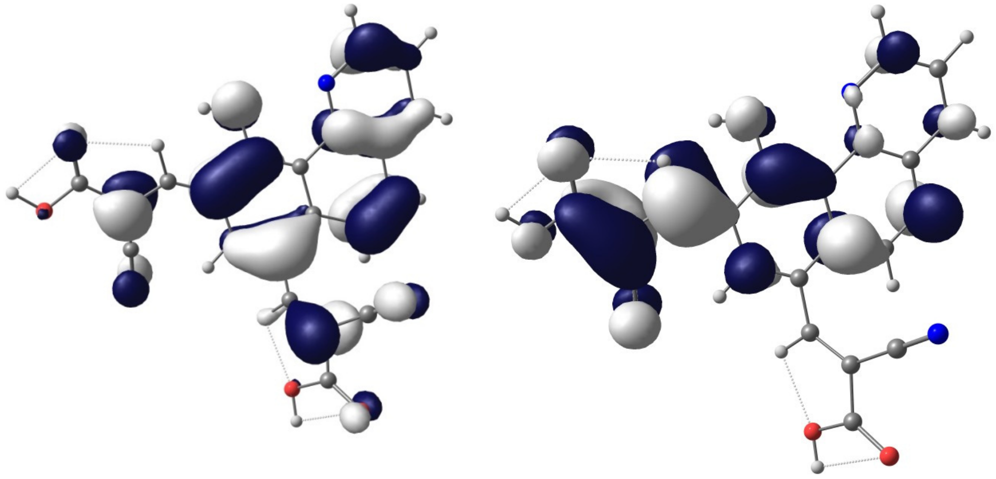

–6.28 eV –3.01 eV

1a

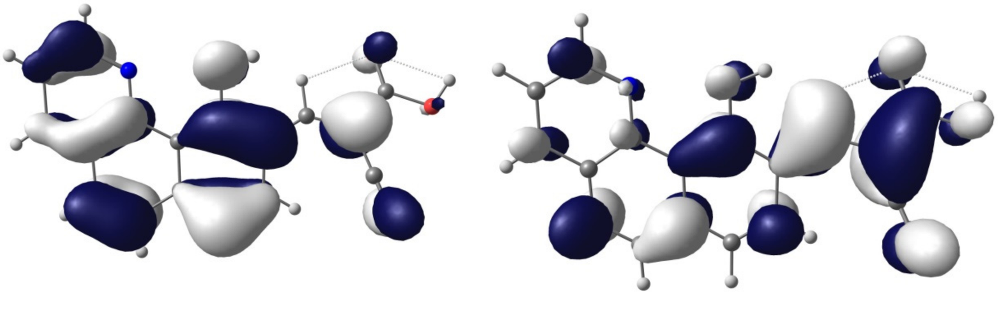

–6.02 eV –2.70 eV

2a-a@Ti21
O54H24

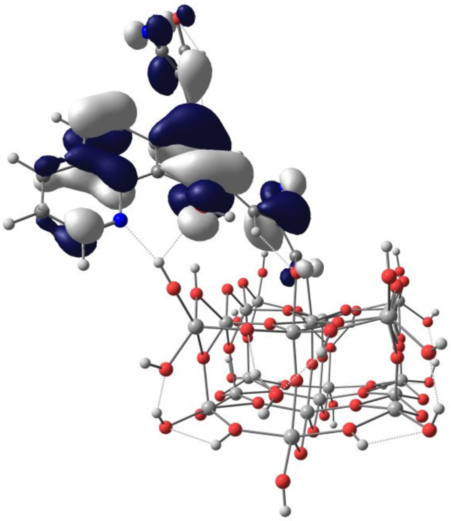

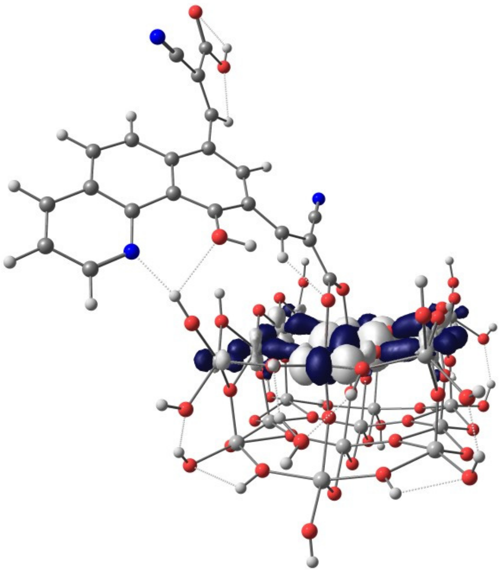

–6.68 eV

–3.60 eV

2a-b@Ti21
O54H24

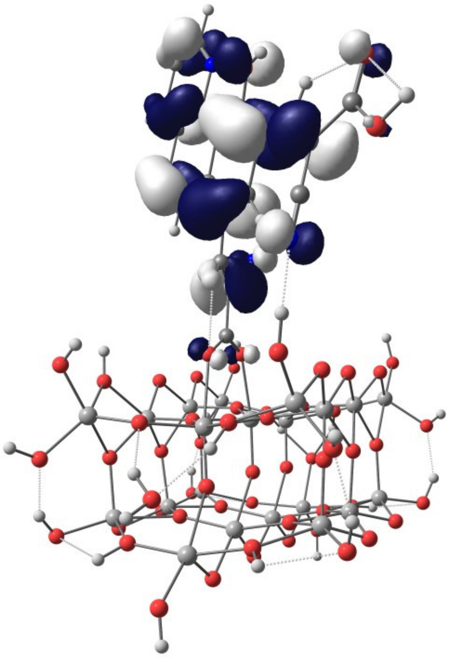

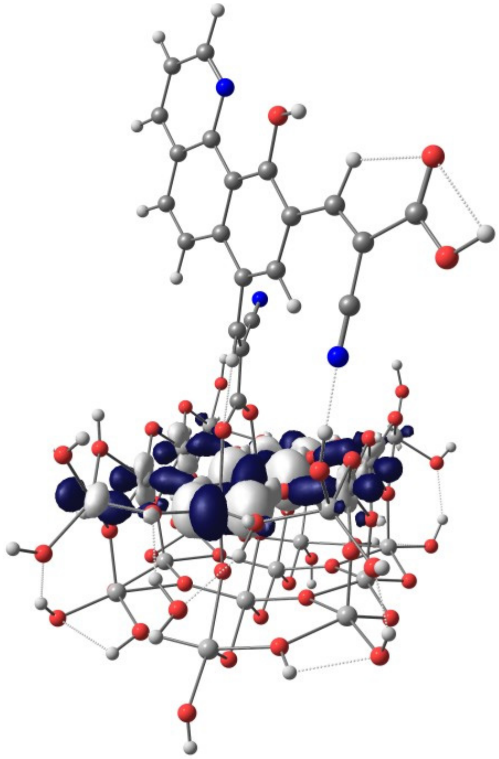

–6.68 eV

–3.61 eV

1a@$Ti_{21}O_{54}H_{24}$

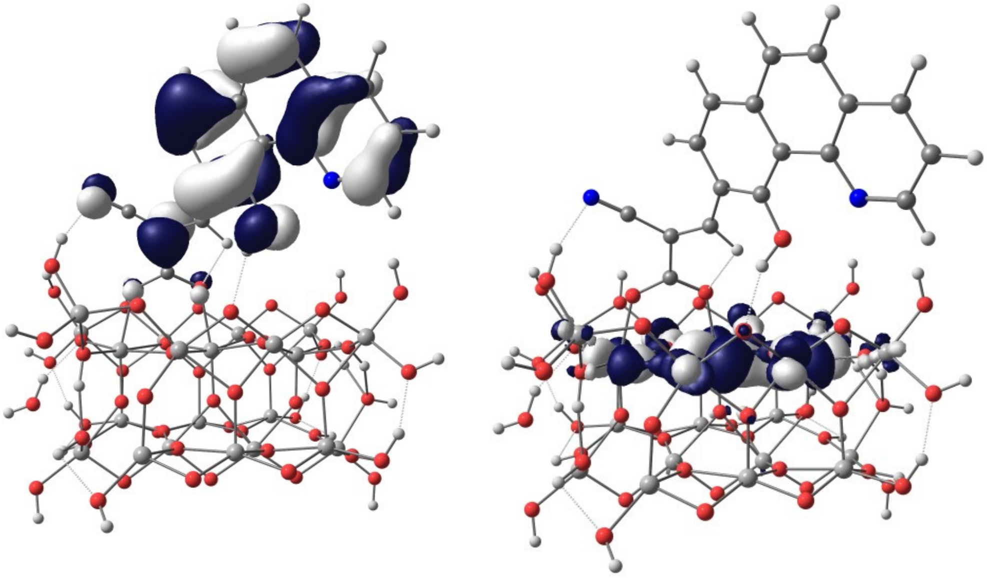

= 9.90 D –5.96 eV –3.60 eV

Figure S12. HOMO and LUMO contours of $Ti_{21}O_{54}H_{24}$, free **1a**, **2a** and adsorbed **1a**/**2a**@$Ti_{21}O_{54}H_{24}$ systems.

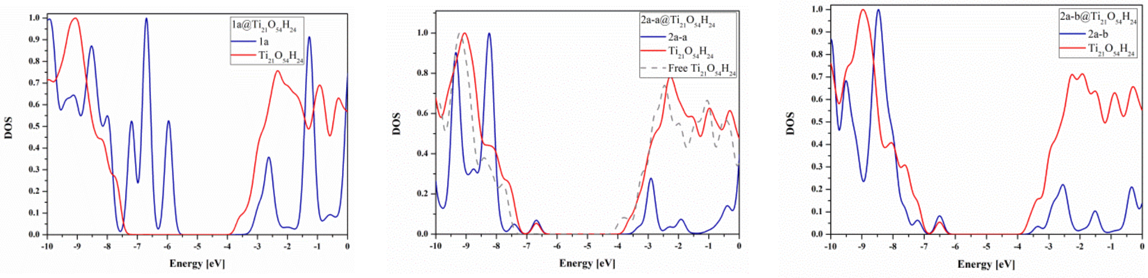

Figure S13. DOS diagrams for **1a**/**2a**@$Ti_{21}O_{54}H_{24}$ systems.

Table S6. Composition of the selected molecular orbitals

2a@$Ti_{21}O_{54}H_{24}$	eV	2a	$Ti_{21}O_{54}H_{24}$	1a@$Ti_{21}O_{54}H_{24}$	eV	1a	$Ti_{21}O_{54}H_{24}$
L+5	−3.04	0	100	L+5	−2.98	3	97
L+4	−3.08	0	100	L+4	−3.03	5	95
L+3	−3.20	2	98	L+3	−3.08	1	99
L+2	−3.30	6	94	L+2	−3.20	1	99
L+1	−3.37	0	100	L+1	−3.36	0	100
LUMO	−3.61	0	100	LUMO	−3.59	0	100
HOMO	−6.68	14	86	HOMO	−5.95	99	1
H−1	−7.41	10	90	H−1	−6.65	100	0
H−2	−7.48	0	100	H−2	−6.73	99	1
H−3	−7.58	0	100	H−3	−7.19	99	1
H−4	−7.63	0	100	H−4	−7.63	0	100
H−5	−7.70	0	100	H−5	−7.66	0	100

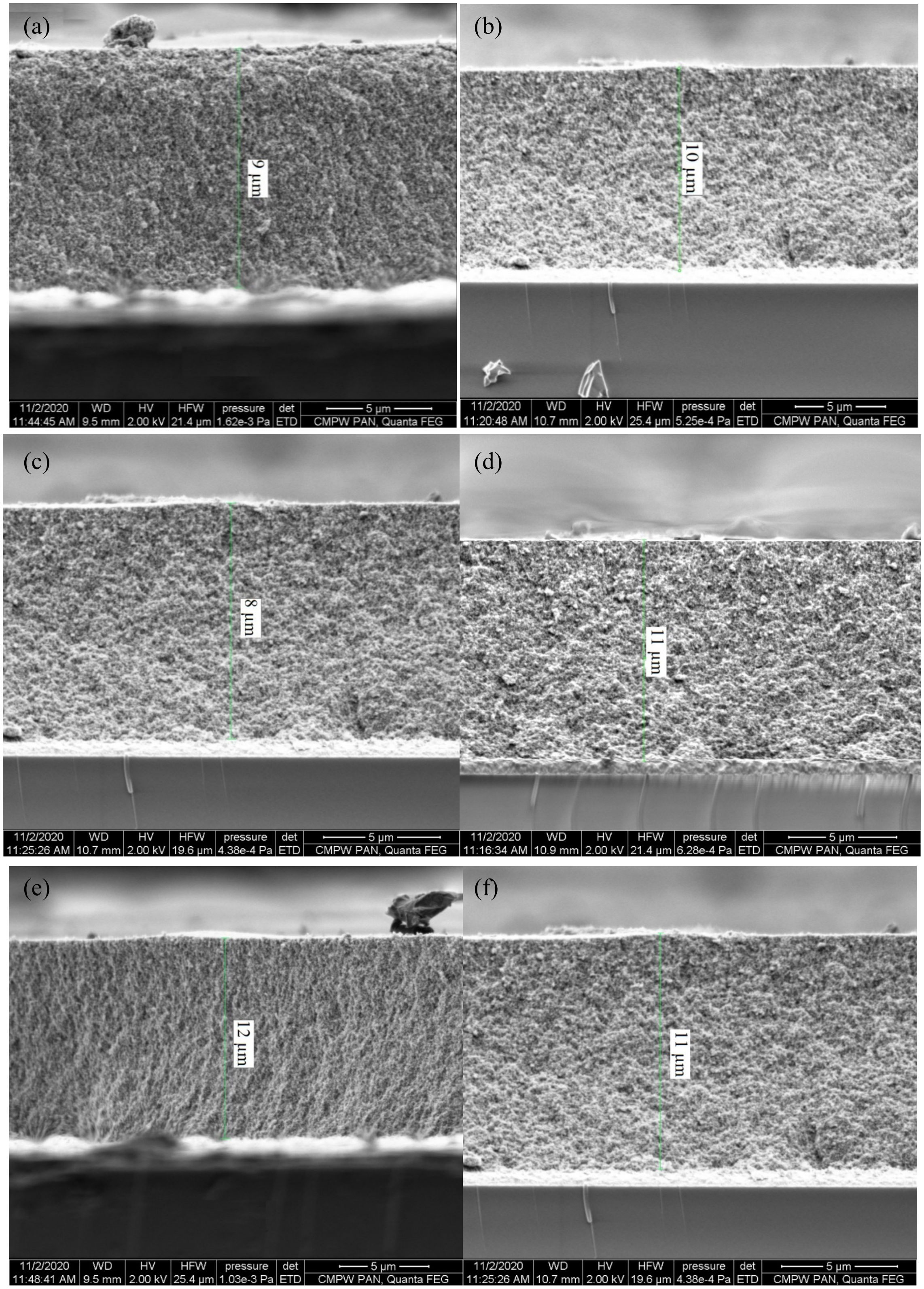

Figure S14. The cross-sectional SEM images of prepared TiO_2 substrates with anchored (**a**) **1a**, (**b**) **2a**, (**c**) **1a**+N719, (**d**) **2a**+N719, (**e**) **1a**+N719+CDCA and (**f**) **2a**+N719+CDCA.

(a)

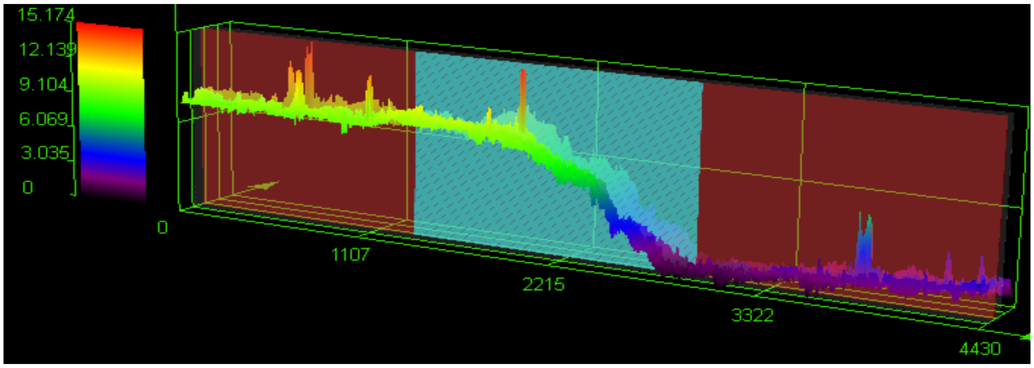
15.174
12.139
9.104
6.069
3.035
0
0
1107
2215
3322
4430

(b)

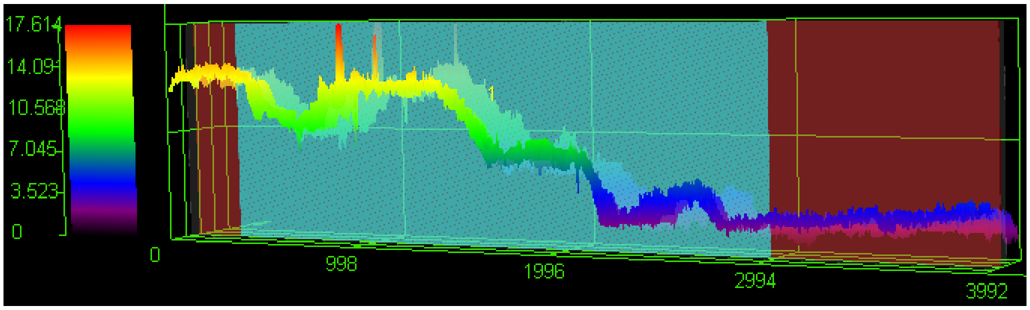
17.614
14.091
10.568
7.045
3.523
0
0
998
1996
2994
3992

(c)

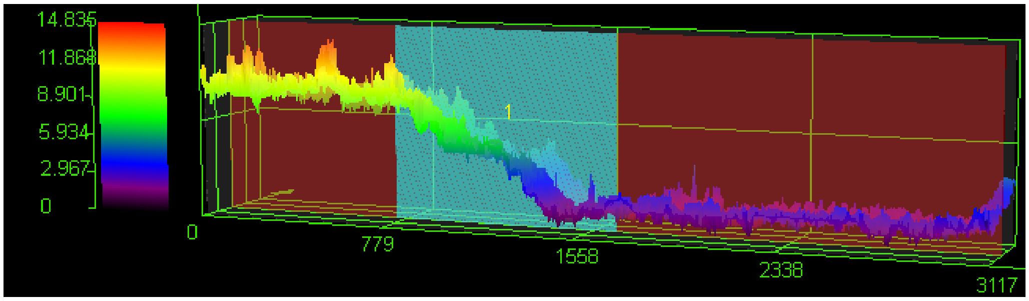
14.835
11.868
8.901
5.934
2.967
0
0
779
1558
2338
3117

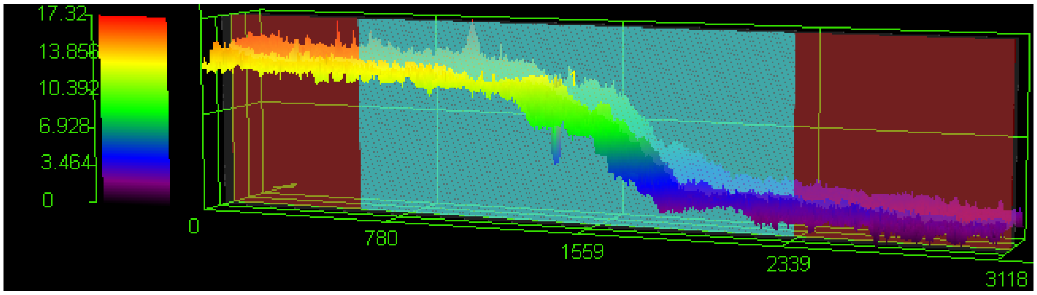
17.32
13.856
10.392
6.928
3.464
0
0
780
1559
2339
3118

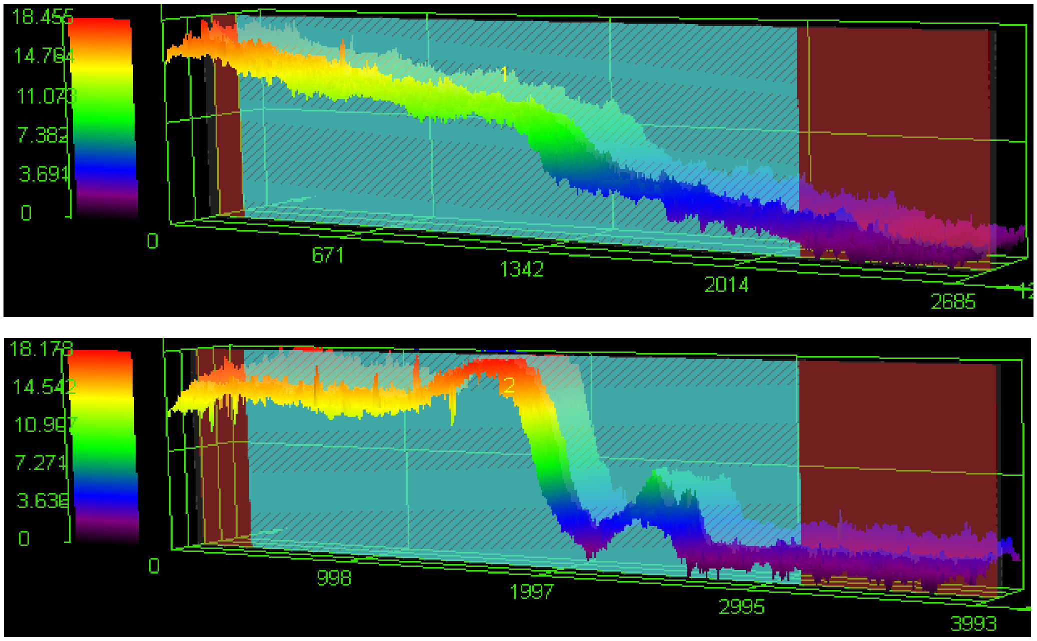

Figure S15. Micrographs of electrodes with (**a**) **1a**, (**b**) **2a**, (**c**) **1a**+N719, (**d**) **2a**+N719, (**e**) **1a**+N719+CDCA and (**f**) **2a**+N719+CDCA using optical microscope.

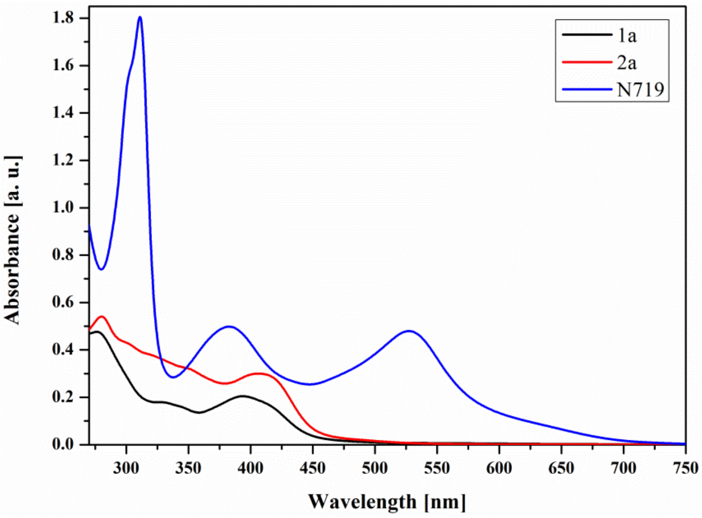

Figure S16. UV-Vis spectra of **1a**, **2a** and N719 in DMF.
